# Relationship Between Fecal Bile Acid Profile and Intestinal Microbiota in Patients With Chronic Radiation Enteritis

**DOI:** 10.1111/1751-2980.70029

**Published:** 2026-01-28

**Authors:** Xin Shen, Song Bo Li, Meng Jie Gao, Jiao Jiao Cao, Hua Yang, Wei Wei Li, Li Chun Wei, Min Chen, Jun Ye Liu, Yong Quan Shi

**Affiliations:** ^1^ State Key Laboratory of Holistic Integrative Management of Gastrointestinal Cancers and National Clinical Research Center for Digestive Diseases, Department of Gastroenterology & Hepatology Xijing Hospital, Fourth Military Medical University Xi'an Shaanxi Province China; ^2^ Postgraduate Department Xi'an Medical University Xi'an Shaanxi Province China; ^3^ Department of Radiation Oncology Xijing Hospital, Fourth Military Medical University Xi'an Shaanxi Province China; ^4^ Department of Radiation Protective Medicine School of Military Preventive Medicine, Fourth Military Medical University Xi'an Shaanxi Province China

**Keywords:** bile acid profile, chronic radiation enteritis, gastrointestinal microbiome, lithocholic acid, uterine cervical neoplasms

## Abstract

**Objective:**

We aimed to investigate the relationship between fecal bile acid (BA) profile and intestinal microbiota in patients with chronic radiation enteritis (CRE).

**Methods:**

Altogether 60 patients with cervical cancer (CC) who visited Xijing Hospital between December 2022 and September 2023 were enrolled, including 20 patients who did not undergo any treatment (the CC group), 20 patients who developed CRE after radical radiotherapy (the CRE group), and 20 patients who did not experience CRE after radical radiotherapy (the non‐CRE [NRE] group). Patients’ characteristics and fecal samples were collected. Fecal BA profiles were quantified, and intestinal microbiota were analyzed by using the 16S rRNA gene sequencing. Differentially expressed BAs and microorganisms were identified across groups, and their correlations were assessed using Spearman's correlation analysis.

**Results:**

In patients with CRE, BA metabolism was characterized by increased proportions of primary BAs and decreased proportions of secondary BAs, particularly lithocholic acid and its isomers. In addition, the abundance of beneficial bacterial genera, such as *Bifidobacterium* and *Megasphaera*, was reduced, whereas that of potentially pathogenic genera, including *Megamonas* and *Dorea*, was increased. Furthermore, a bidirectional relationship between BA metabolism and intestinal microbiota was observed.

**Conclusions:**

Patients with CRE present notable alterations in BA metabolism and intestinal microbiota. CRE may trigger a harmful feedback mechanism driven by the interaction between these two factors. Targeted regulation of BA metabolism and intestinal microbiota may be a promising therapeutic approach for the management of CRE.

**Trial Registration:** ClinicalTrials.gov identifier: NCT05728060.

## Graphical abstract

Patients with chronic radiation enteritis (CRE) demonstrate dysregulated bile acid (BA) metabolism, characterized by an increased proportion of primary BAs and a decreased proportion of secondary BAs. Moreover, the diversity of the intestinal microbiota is significantly reduced in individuals with CRE, and there is a bidirectional interaction between BA metabolism and gut microbial composition in patients with CRE. CC, cervical cancer; NRE, non‐CRE; UPLC‐MS/MS, ultra‐performance liquid chromatography‐tandem mass spectrometry.

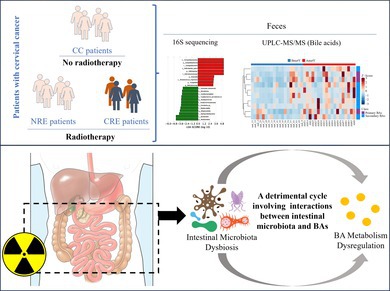

## 1 Introduction

Owing to the increasing incidence of pelvic malignancies and the improvement in radiation technologies, radiotherapy has been increasingly applied in clinical practice, leading to a rise in its related complications [1, 2]. Radiotherapy enhances survival outcomes among cancer patients; however, it stimulates oxidative stress via the production of reactive oxygen species, which may damage normal tissues, particularly the gastrointestinal (GI) epithelial cells that have a high replication rate, resulting in radiation enteritis (RE) [3, 4, 5, 6]. Acute radiation enteritis (ARE), which typically resolves spontaneously, can often be effectively prevented and managed with medications and advanced radiotherapy protocols. However, if symptoms and signs of ARE persist for more than 3 months or recur within 3 months after radiotherapy, it may progress to chronic radiation enteritis (CRE), which greatly affects patient's quality of life [7, 8]. To date, the underlying mechanisms of CRE remain largely undefined, and currently available treatments are limited and yield suboptimal outcomes. Consequently, the prevention of CRE has become a critical priority in clinical setting.

RE is known to be closely associated with intestinal microbiota. The diversity and composition of intestinal microbiota are altered following radiotherapy. A previous study has shown that germ‐free mice present significant resistance to radiation‐induced intestinal damage [9]. In addition, antibiotic pre‐treatment in mice facilitates the reconstitution of intestinal microbiota after radiation exposure and alleviates intestinal inflammation and prevents intestinal fibrosis [10]. Moreover, probiotics and fecal microbiota transplantation (FMT) can mitigate RE by restoring microbial equilibrium [11]. Further research has demonstrated that the protective effect of intestinal microbiota modulation on RE is primarily mediated through small‐molecule substances, such as microbial metabolites [12, 13]. These metabolites include bile acids (BAs), short‐chain fatty acids (SCFAs), and tryptophan metabolites, among others. BAs are key metabolic products that influence intestinal microbiota and host health via the inhibition of pathogenic bacteria and maintenance of the intestinal mucosal barrier. Disorders of BA metabolism, particularly those of secondary BAs, may be associated with irradiation‐induced intestinal injury [14, 15]. Current research on BAs and RE predominantly focuses on acute radiation‐induced intestinal injury in rodent models. However, the prevalence of CRE 10 years after irradiation ranges from 10% to 20%, exerting long‐term adverse effects on patients' daily lives [7]. Therefore, the interaction between BAs and intestinal microbiota in patients with CRE warrants further investigation.

Given the key role of intestinal microbiota in CRE, therapeutic benefits may be achieved by investigating their relationship with bile BA profiles and modulation. In this study, we recruited untreated cervical cancer (CC) patients, patients who developed CRE following radiotherapy, and those who did not develop CRE after radiotherapy to analyze the differences in their intestinal microbial composition and fecal BA profiles, and to explore the relationship between fecal BA profiles and intestinal microbiota in patients with CRE.

## 2 Patients and Methods

### 2.1 Study Design and Participants

CC patients aged 18–75 years who were scheduled to receive or had already undergone radical radiotherapy in the absence of surgery at Xijing Hospital, Fourth Military Medical University (Xi'an, Shaanxi Province, China) between December 2022 and September 2023 were prospectively evaluated for eligibility. The exclusion criteria were as follows: (i) patients with diseases that might affect BA metabolism, such as hepatitis, liver cirrhosis, and gallstone disease; (ii) patients who had previously undergone cholecystectomy or partial resection of any part of the GI tract; (iii) those who were treated with medications that might influence BA metabolism during the past 30 days before their enrollment, such as ursodeoxycholic acid (UDCA) and obeticholic acid; (iv) those who had consumed probiotics or antibiotics within 30 days before their enrollment; (v) those with abnormal liver function, defined as alanine aminotransferase (ALT) level exceeding twice the upper limit of normal (ULN), moderate‐to‐severe renal dysfunction (creatinine [Cr] > 177 mmol/L), or moderate‐to‐severe chronic obstructive pulmonary disease; (vi) those with mental or legal disabilities; (vii) those with a previous history of alcohol or drug abuse; and (viii) those who could not fully understand the content of or voluntarily sign the informed consent, or declined follow‐up examinations.

Finally, 60 patients met the eligibility criteria and were included in the study. They were further evenly divided into three groups: (i) the case group (the CRE group), consisting of 20 patients who developed CRE after radical radiotherapy for CC; (ii) the control group A (the non‐CRE [NRE] group), including 20 patients who received radical radiotherapy for CC but did not experience CRE; and (iii) the control group B (CC group), comprising 20 patients with CC who did not undergo any treatment.

This study was approved by the Ethics Committee of Xijing Hospital, Fourth Military Medical University (no. KY20222208‐F‐1) and was conducted in accordance with the Declaration of Helsinki (Japan, 1975) and its later amendments. The trial was registered on ClinicalTrials.gov (identifier no. NCT05728060). Written informed consent was obtained from each participant before their enrollment.

### 2.2 Diagnosis of CC and CRE

CC were diagnosed based on pathological findings of the lesions, and its staging was conducted in accordance with the 2018 FIGO Gynecologic Oncology Committee staging system [16]. In addition, the diagnostic criteria for CRE were as follows [17]: (i) a previous history of radiotherapy for cervical tumor, presenting with symptoms or signs after 3 months since the completion of radiotherapy with or without chemotherapy; (ii) clinical manifestations including hematochezia, increased frequency of bowel movements, bowel urgency, mucoid stool, abdominal pain, tenesmus, intestinal stenosis, perforation, or fistula formation; and (iii) endoscopic and pelvic imaging findings that were consistent with CRE. The diagnosis of CRE was made only after other potential etiologies, including acute infectious enteritis, inflammatory bowel disease, tumor metastasis or recurrence, and amoebic enteritis, had been systematically ruled out.

### 2.3 Fecal Sample Collection

Fecal samples were collected from all participants after they were fasted overnight. The samples were collected from the central area of the feces and stored in sterile fecal collection tubes (5 mL), and were divided into two portions and kept in a refrigerator at −80°C for subsequent unified 16S rRNA sequencing and BA profiling.

### 2.4 BA Profiling and Quantitation

BA profiling of the fecal samples was carried out by using ultra‐performance liquid chromatography‐tandem mass spectrometry (UPLC‐MS/MS; Metabo‐Profile Inc., Shanghai, China), as described previously [18]. The raw data files were then processed using the MassLynx software (Waters, Milford, MA, USA), which included peak integration as well as calibration and quantification of each metabolite. Statistical analyses were performed using the R software version 3.5.3 (R Foundation for Statistical Computing, Vienna, Austria).

### 2.5 16S rRNA Gene Sequencing Analysis

To characterize the bacterial microecology in the fecal samples, 16S rRNA gene sequencing was used. Sequencing libraries were constructed using the TruSeq Nano DNA LT Library Preparation Kit (Illumina, San Diego, CA, USA). Effective tag denoising, species annotation, and community diversity analyses were conducted using the Quantitative Insights Into Microbial Ecology 2 (QIIME2) software version 2019.4 (University of California San Diego, San Diego, CA, USA). Cluster analysis, principal coordinate analysis (PCoA), and differential species screening were conducted using the R software version 3.5.3 (R Foundation for Statistical Computing). The linear discriminant analysis effect size (LEfSe) analysis was conducted using a combination of tools, including Python (version 3.7; Python Software Foundation, Wilmington, DE, USA) LEfSe package, and the R packages ggplot2 and ggtree, to identify potential biomarkers for CRE.

### 2.6 Statistical Analysis

Data processing and all the statistical analyses were performed using SPSS Statistics version 26.0 (IBM, Armonk, NY, USA), R software version 3.5.3 (R Foundation for Statistical Computing), and the iMAP software version 1.0 (Metabo‐Profile Inc.). Continuous variables were presented as median and interquartile range (IQR). The distribution of BAs was assessed using the Shapiro–Wilk test. Comparisons of continuous data were performed by using the paired *t*‐test or nonparametric statistical methods, depending on data normality. Categorical variables were presented as numbers and percentages or frequencies, with comparisons using the Chi‐square or Fisher's exact test, when appropriate. To elucidate the differences between groups, the orthogonal partial least squares discriminant analysis (OPLS‐DA) was used to investigate the distribution pattern of fecal BA profiling. The Spearman's correlation analysis was performed on the significantly different BAs and fecal microbiota at different classification levels in each group, and the BAs and intestinal bacteria with correlations (correlation coefficient > 0.3, *p* < 0.05) were identified. A *p* value of less than 0.05 was regarded as statistically significant.

## 3 Results

### 3.1 Demographics of the Enrolled Participants

A total of 60 patients were ultimately included in the study, including 20 patients with CC who did not undergo any treatment, 20 patients who received radiotherapy for CC and developed CRE, and 20 patients who underwent radiotherapy for CC but did not experience CRE. The external pelvic irradiation dose for both the NRE and CRE groups was 50 Gy, delivered in fractions of 2.0 Gy each. On average, the time duration from the completion of radiotherapy to the onset of hematochezia in the CRE group was 8.9 months. A statistically significant difference was observed between the NRE group and the CRE group regarding the type of radiotherapy used, further substantiating that intensity‐modulated radiation therapy (IMRT) effectively reduced the incidence of CRE compared to three‐dimensional conformal radiotherapy (3DCRT) (65.0% vs. 30.0%, *p* < 0.05). However, no statistically significant differences were observed in patients' age, body mass index (BMI), clinical stage of CC, previous history of hypertension, diabetes mellitus, or coronary heart disease between the CRE group and either the NRE group or the CC group (all *p* > 0.05; Table 1).

**TABLE 1:** Characteristics of the study participants in the chronic radiation enteritis (CRE), non‐CRE (NRE), and cervical cancer (CC) groups.

Characteristics	CRE group (*n* = 20)	Control groups	
		NRE group (*n* = 20)	CC group (*n* = 20)
Age, years (mean ± SD)	59.85 ± 8.09	54.55 ± 10.31	57.35 ± 8.18
BMI, kg/m^2^ (mean ± SD)	23.31 ± 2.63	22.87 ± 2.04	23.90 ± 3.27
Marital status (married) (*n*, %)	20 (100)	20 (100)	20 (100)
Clinical stage of CC (*n*, %)			
Stage I	0 (0)	1 (5.0)	0 (0)
Stage II	5 (25.0)	2 (10.0)	5 (25.0)
Stage III	13 (65.0)	17 (85.0)	13 (65.0)
Stage IV	2 (10.0)	0 (0)	2 (10.0)
Radiotherapy (*n*, %)			
IMRT	6 (30.0)	13 (65.0)*	0 (0)
3DCRT	14 (70.0)	7 (35.0)	0 (0)
History of hypertension (no) (*n*, %)	19 (95.0)	16 (80.0)	20 (100)
History of coronary heart disease (no) (*n*, %)	19 (95.0)	18 (90.0)	20 (100)
History of diabetes mellitus (no) (*n*, %)	18 (90.0)	20 (100)	20 (100)

Abbreviations: 3DCRT, three‐dimensional conformal radiotherapy; BMI, body mass index; IMRT, intensity‐modulated radiation therapy; SD, standard deviation.

*p < 0.05 compared with the CRE group.

### 3.2 Differences in Fecal BA Profiles Between the CRE and the Control Groups

Altogether 41 types of BAs were detected in the fecal samples of the patients (Table S1). Notably, the average concentration of total BAs was significantly elevated in both the CRE group and the NRE group compared to the CC group (*p* < 0.05). Primary BAs constituted 39.9%, 53.6%, and 42.1% of the total BAs, respectively, while secondary BAs constituted 60.1%, 46.4%, and 57.9% of the total BAs in the CC, NRE, and CRE groups (Figure S1a). The partial least squares discriminant analysis (PLS‐DA) model showed that fecal BA profiles significantly differed among the three groups (Figure S1b). Notably, levels of 31 BAs exhibited significant differences among the three groups. The levels of ω‐muricholic acid (ωMCA), β‐cholic acid (β‐CA), 12‐dehydrocholic acid (12‐DHCA), 3‐dehydrocholic acid (3‐DHCA), 7‐ketolithocholic acid (7‐KetoLCA), 7,12‐diketolithocholic acid (7,12‐DiketoLCA), and 6‐ketolithocholic acid (6‐KetoLCA) in both the NRE and CRE groups significantly increased compared to the CC group, while those of lithocholic acid (LCA) and isolithocholic acid (isoLCA) decreased (Figure S1c). Moreover, significant differences were observed in the ratios of deoxycholic acid (DCA) to cholic acid (CA), CA to chenodeoxycholic acid (CDCA), and LCA to CDCA among the groups (all *p* < 0.05). This indicated that BA metabolism in patients who had undergone radiotherapy for CC was significantly disrupted.

**FIGURE 1:**
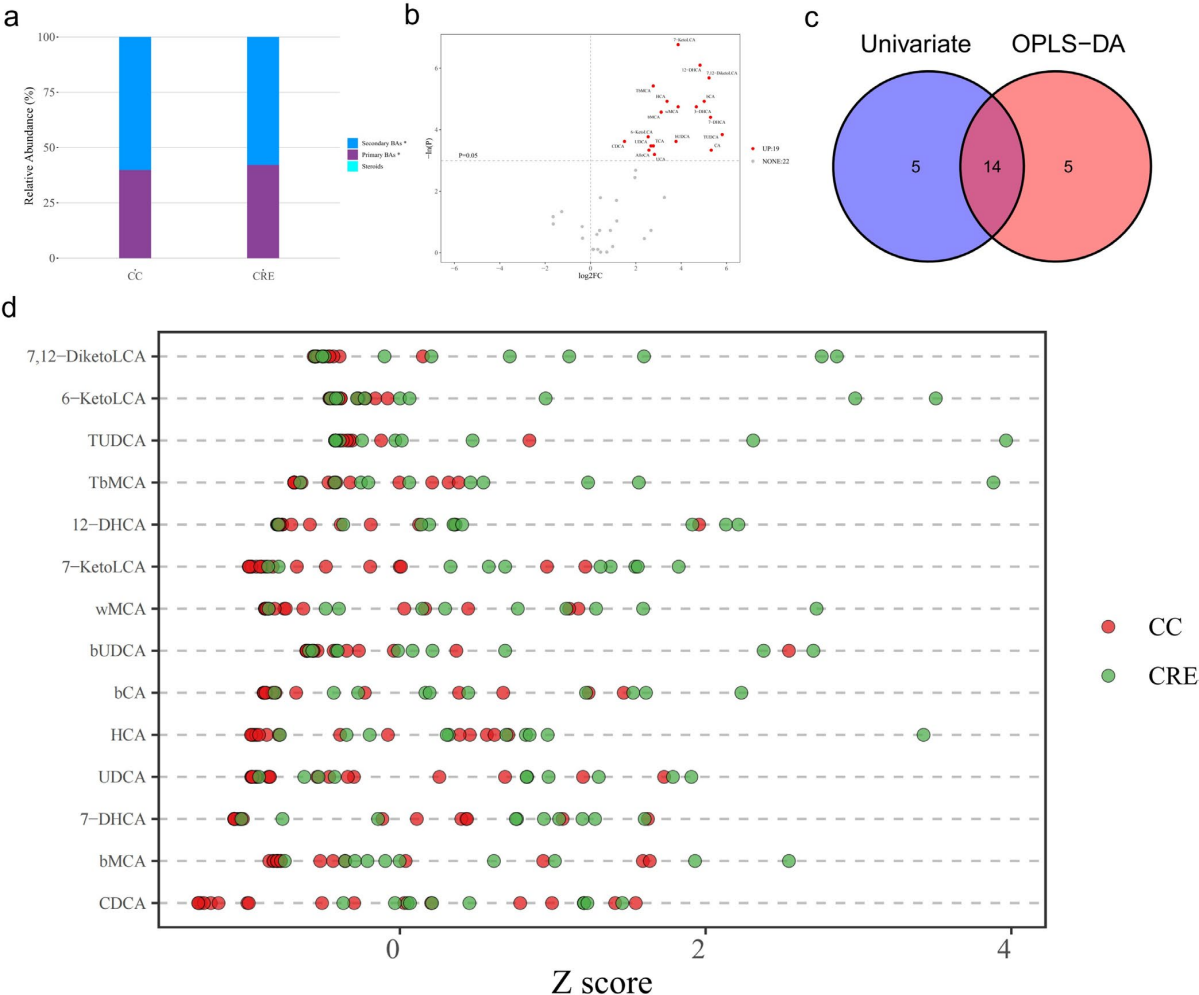
Altered bile acid (BA) profiles in the chronic radiation enteritis (CRE) group compared with the cervical cancer (CC) control group. (a) Stacked bar chart of the relative proportions of primary and secondary BAs between the two groups. (b) Volcano plot of the differential BAs in CRE filtered by univariate analysis. (c) Venn plot of the differential BAs filtered by the orthogonal partial least squares discriminant analysis (OPLS‐DA) model and the univariate analysis. (d) *Z*‐score dot plot of the 14 differential BAs between the two groups. NONE, metabolites that fail to meet the screening criteria; UP, upregulated. *p < 0.05.

**FIGURE 2:**
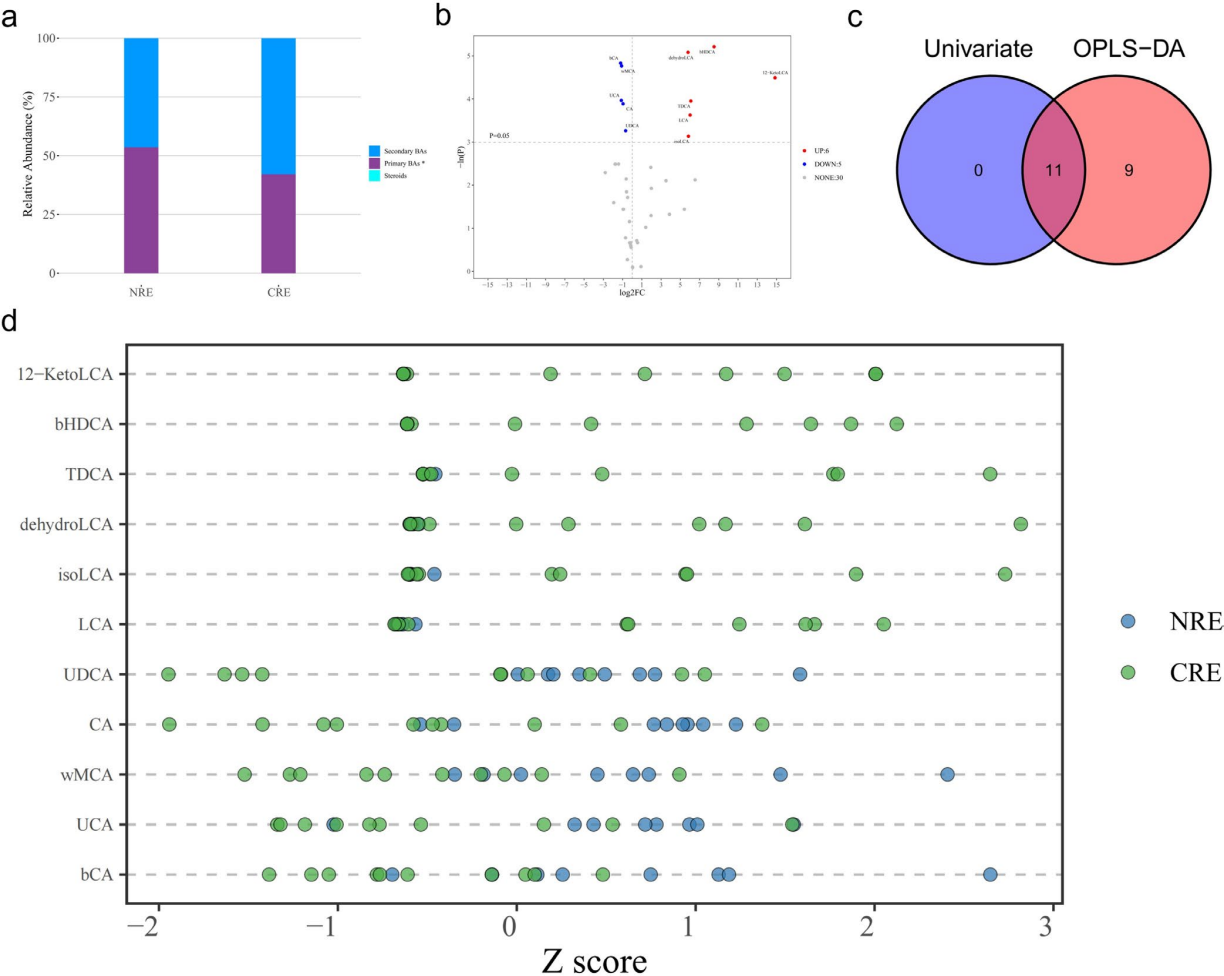
Altered bile acid (BA) profiles in the chronic radiation enteritis (CRE) group compared with the non‐CRE (NRE) group. (a) Stacked bar chart of the relative proportions of primary and secondary BAs between the two groups. (b) Volcano plot of the differential BAs in CRE versus NRE by using the univariate analysis. (c) Venn plot of the differential BAs filtered by the orthogonal partial least squares discriminant analysis (OPLS‐DA) model and univariate analysis. (d) Z‐score dot plot of the 11 differential BAs between the two groups. DOWN, downregulated; NONE, metabolites that fail to meet the screening criteria; UP, upregulated. *p < 0.05.

The profiles of primary and secondary BAs significantly differed between the CRE group and the CC group (Figure 1a). In addition, significant differences were also observed in the levels of primary BAs between the NRE group and the CRE group (Figure 2a). To maximize the identification of differential metabolites in patients with CRE, an OPLS‐DA model was constructed. We found that the fecal BA profile in CRE patients was separate from those of the CC (Figure S2a) and NRE (Figure S2b) patients. The permutation test was performed to ensure that the model was not overfitted (Figure S2c,d).

Of the 41 BAs detected, 19 of them contributed significantly to the distinguishment of CRE and CC, with a variable importance in projection (VIP) value of > 1. Using the Mann–Whitney *U*‐test, there were also 19 BAs found to be significantly changed (Figure 1b). As a result, the 14 overlapping BAs were considered as differential BAs in CRE patients (Figure 1c). Furthermore, levels of the 14 BAs were all increased in the CRE group compared to the CC group, including 7‐KetoLCA, 12‐DHCA, 7,12‐DiketoLCA, taurine‐β‐muricholic acid (TbMCA), β‐CA, and hyocholic acid (HCA) (Figure 1d).

In addition, 20 BAs contributed significantly in distinguishing between CRE and NRE, with a VIP value of > 1. The subsequent Mann–Whitney *U*‐test showed that 11 BAs were significantly changed (Figure 2b). Therefore, these 11 overlapping BAs were considered as differential BAs in CRE patients when compared with the NRE patients (Figure 2c). Of these, the levels of five BAs were decreased in CRE, namely β‐CA, ωMCA, ursocholic acid (UCA), CA, and UDCA. In contrast, those of the other six BAs, that is, β‐hyodeoxycholic acid (bHDCA), dehydrolithocholic acid (dehydroLCA), 12‐ketolithocholic acid (12‐KetoLCA), LCA, taurodeoxycholic acid (TDCA), and isoLCA, were significantly increased in CRE (Figure 2d).

### 3.3 Differences in Fecal Microbiota Between the CRE and the Control Groups

The α‐diversity was used to assess the microbial community diversity in the fecal samples of the patients. The Observed_species, Simpson, and Chao1 indexes in both the CRE and NRE groups showed a decreasing trend (Figure S3a) compared to the CC group. Although the differences were not statistically significant, these results suggested a reduction in the number of intestinal microbial species in patients undergoing radiotherapy, indicating a potential decline in both the diversity and evenness of the microbial community. The β‐diversity was then used to assess the differences in microbial composition across distinct microbial communities by employing PCoA and non‐metric multidimensional scaling (NMDS). In the PCoA plot, the distances between the three groups were relatively small, indicating high similarity in their microbial community composition and species abundance (Figure S3b). The NMDS plot revealed a stress value below 0.2, suggesting a reliable ordination (Figure S3c).

**FIGURE 3:**
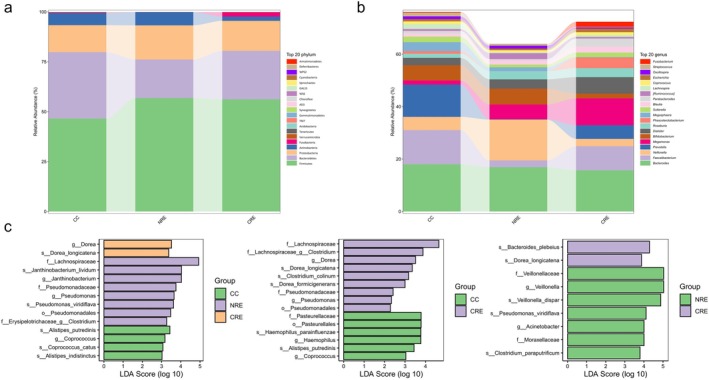
Altered intestinal microbiota in the cervical cancer (CC), chronic radiation enteritis (CRE), and non‐CRE (NRE) groups. (a) The top 20 most abundant phyla in each group were selected to construct a relative abundance bar chart. (b) The top 20 most abundant genera in each group were selected to construct a relative abundance bar chart. (c) The linear discriminant analysis (LDA) effect values of the indicator species in each group.

Based on the species annotation results, the top 20 most abundant phyla in each group were selected to construct a relative abundance bar chart (Figure 3a). The dominant phyla across all three groups were Firmicutes, Bacteroidetes, Proteobacteria, and Actinobacteria. The relative abundances of Firmicutes and Proteobacteria were increased in both the CRE and NRE groups compared to the CC group, whereas that of Bacteroidetes was decreased. In comparison with the NRE group, the CRE group exhibited a higher relative abundance of Bacteroidetes but a lower relative abundance of Actinobacteria. In addition, at the genus level, the CRE group showed reduced relative abundances of *Bifidobacterium* and *Megasphaera* compared to both the CC group and the NRE group. In contrast, the relative abundances of *Megamonas*, *Dialister*, *Phascolarctobacterium*, and *Roseburia* were elevated in the CRE group compared to the two control groups (Figure 3b).

The LEfSe analysis identified distinct marker species that were specific to each group (linear discriminant analysis score > 2, *p* < 0.05). Compared with the CC group, the CRE group exhibited higher abundances of *f_Lachnospiraceae*, *f_Lachnospiraceae_g_Clostridium*, *g_Dorea*, *s_Dorea_longicatena*, *s_Clostridium_colinum*, *s_Dorea_formicigenerans*, *f_Pseudomonadaceae*, *g_Pseudomonas*, and *o_Pseudomonadales*, while the CC group showed higher abundances of *f_Pasteurellaceae*, *o_Pasteurellales*, *s_Haemophilus_parainfluenzae*, *g_Haemophilus*, *s_Alistipes_putredinis*, and *g_Coprococcus* (Figure 3c).

### 3.4 Association Between Fecal BA Profiles and Intestinal Microbiota

**FIGURE 4:**
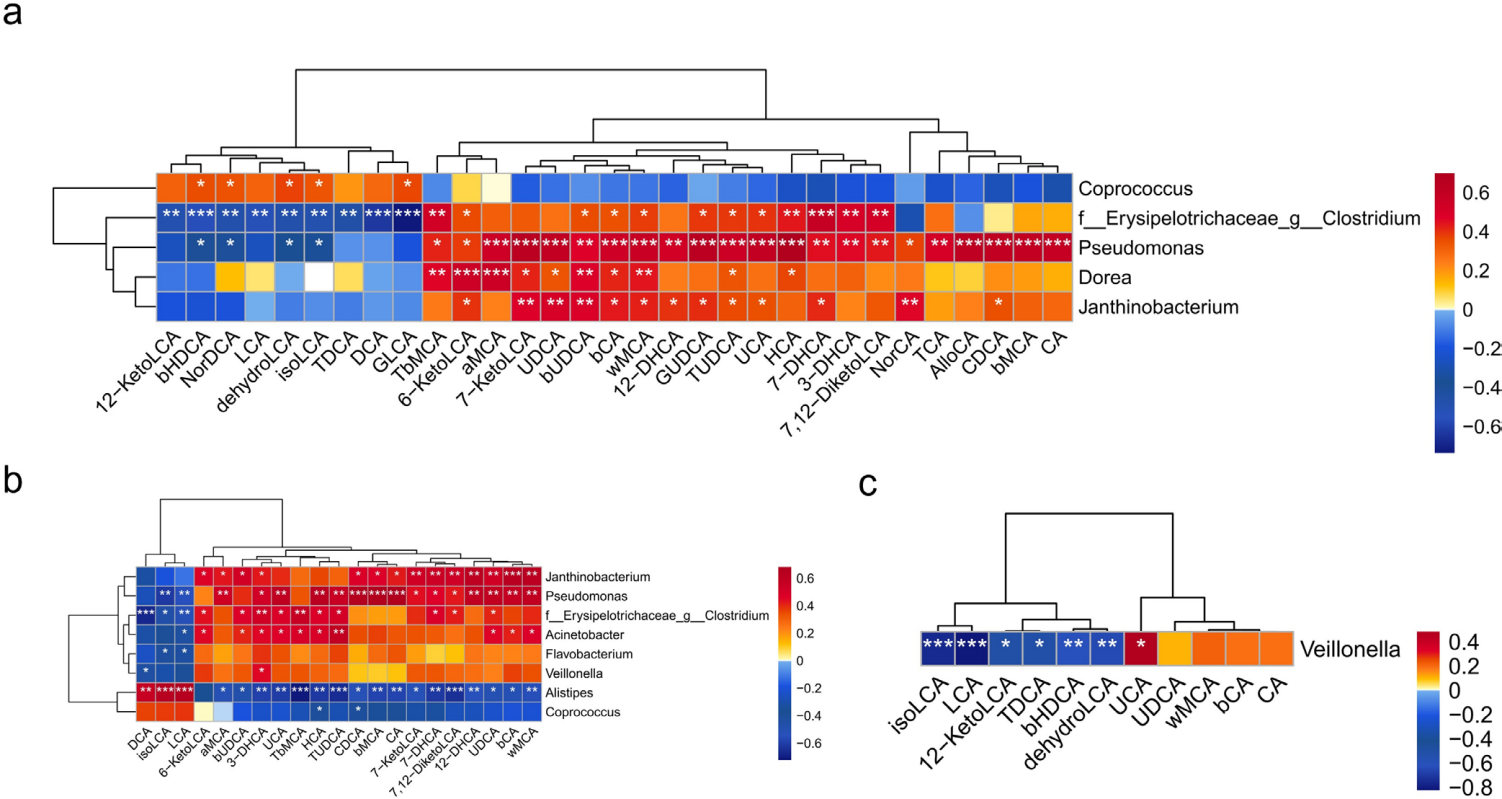
Correlation between fecal bile acid (BA) profiles and intestinal microbiota (at the genus level) (a) among the chronic radiation enteritis (CRE), non‐CRE (NRE), and cervical cancer (CC) groups, (b) in the CC group and the CRE group, and (c) in the NRE and CRE groups. *p < 0.05, **p < 0.01, ***p < 0.001.

Following the analysis of the association between fecal BA profiles and intestinal microbiota across the three groups, we found that *Pseudomonas*, *Veillonella*, *f_Erysipelotrichaceae_g_Clostridium*, and other microbial taxa exhibited similar correlation patterns with BA profiles, showing positive correlations with various BA derivatives but negative correlations with isoLCA and LCA. In addition, they were somewhat more abundant in patients with CRE. In contrast, *Alistipes* demonstrated a positive association with isoLCA and LCA levels (Figure 4). Furthermore, our findings revealed that the genus *Dorea*, which was enriched in the intestinal microbiota of CRE patients, showed a positive correlation with BA metabolites such as TβMCA, 6‐KetoLCA, α‐MCA, and 7‐KetoLCA.

## 4 Discussion

In this study, we examined the fecal BA profiles and intestinal microbiota in patients with CRE, NRE, and those with CC but did not undergo radiation therapy, and found notable differences in both fecal BA profiles and intestinal microbiota among the three groups. Furthermore, we identified a significant relationship between fecal BA profiles and intestinal microbial composition in CRE cases.

The varied profiles of BAs across the three groups in our study suggest that BA metabolism is disrupted in CRE cases, which may potentially result from radiation‐induced injury that impairs the enterohepatic circulation of BAs [19]. Our study found that the concentrations of primary BAs in fecal samples of patients undergoing radiotherapy were elevated compared to those who never underwent any therapy, which might be due to impaired BA reabsorption and a reduced bacterial capacity for metabolic transformation. Intestinal microbiota dysbiosis, particularly a decrease in microorganisms possessing bile salt hydrolase (BSH) and 7α‐dehydroxylase activity, has been reported to be associated with diminished efficiency in converting primary BAs into secondary BAs [20]. Consequently, the production of secondary BAs was significantly reduced, which aligns with our findings. Our previous study had shown that the relative abundance of secondary BAs decreased while that of primary BAs increased in irradiated mice, with LCA showing the most significant change, which potentially alleviated intestinal inflammation [15]. In the current study, however, we found that compared with the CC group, in the NRE and CRE groups both LCA and isoLCA abundances were markedly decreased.

Radiation‐induced intestinal injury directly disrupts the integrity of intestinal mucosal barrier, alters intestinal microenvironment, and ultimately results in a significant decrease in overall microbial diversity. On one hand, the abundance of beneficial gut bacteria—particularly those that produce SCFAs—reduces. SCFAs serve as a key energy source for intestinal epithelial cells, playing a crucial role in maintaining the intestinal barrier, reducing inflammation, and regulating immune function [21, 22]. The relative abundance of *Bifidobacterium* and *Megasphaera* in the CRE group was observed to be lower than that in both the CC group and the NRE group. *Bifidobacterium longum* has been shown to effectively alleviate colitis in mice and may serve as a potential alternative or adjunct for the treatment of inflammatory bowel disease (IBD) [23]. Branched SCFA‐rich fermented protein food was found to enhance the growth performance and intestinal health by increasing colonic isobutyric and isovaleric acid levels and promoting the abundance of potential probiotics such as *Megasphaera* in porcine models [24]. Randomized clinical trials have demonstrated that probiotic preparations, including *Bifidobacterium* and *Lactobacillus* species, are effective in reducing both the incidence and severity of diarrhea induced by radiotherapy [25, 26]. FMT has shown therapeutic potential in managing CRE, with patients exhibiting intestinal microbiota alterations resembling those of the donors following the procedure [27, 28, 29]. On the other hand, the abundance of (potentially opportunistic) pathogenic bacteria increases. Our study revealed that the genus *Megamonas* was significantly enriched in the CRE group. As a core member of the intestinal microbiota, it may represent a microbial signature specific for the Asian population and is closely associated with diseases such as IBD [30] and colorectal cancer [31]. In addition, the genus *Dorea* was markedly increased in the CRE group as well, which has also been observed in patients with diarrhea‐predominant irritable bowel syndrome [32].

CRE can initiate a detrimental cycle involving the interaction between intestinal microbiota and BAs. First, imbalance in the intestinal microbial composition results in the dysregulation of BA metabolism. We observed a significant increase in the abundance of the genus *Dorea* in the CRE group, which exhibited positive correlations with TβMCA, 6‐KetoLCA, α‐MCA, and 7‐KetoLCA. Previous studies have suggested that the genus *Dorea* is associated with alterations in BA metabolism and the enzymatic activities within the BA metabolic pathway [33]. *Pseudomonas* has been shown to influence the biotransformation of LCA [34], which aligns with our results. Second, disorders of BAs contribute to the exacerbation of gut dysbiosis and inflammatory responses. Elevated levels of primary BAs exert cytotoxic and pro‐inflammatory effects on intestinal epithelial cells and disrupt the intestinal microbiota composition, which is positively associated with the presence of potentially pathogenic bacterial taxa, including Proteobacteria, *Escherichia coli* , and Actinobacteria [35]. Previous studies have indicated that centenarians possess distinct secondary BAs, including several isoforms of LCA, such as iso‐, 3‐oxo‐, allo‐, 3‐oxoallo‐, and isoalloLCA. Among these compounds, isoalloLCA has demonstrated significant antimicrobial activity specifically against Gram‐positive multidrug‐resistant pathogens such as *Clostridioides difficile* and *Enterococcus faecium* [36]. This study also showed a significant increase in *Alistipes* after incubation with isoalloLCA compared to other BA compounds. This is consistent with the higher abundance of *Alistipes* observed in the intestinal microbiota of centenarians, suggesting that isoalloLCA can directly influence intestinal microbial composition [36]. Our study also found a positive correlation between isoLCA and LCA levels and the presence of *Alistipes*, while a negative correlation was observed with *Pseudomonas*, a bacterium that has been consistently found to be enriched in the intestine of CRE individuals.

Previous studies have predominantly focused on BA profiles with colorectal cancer and IBD. Cai et al. found that primary BAs were overabundant whereas secondary BAs were reduced in IBD, which is consistent with our findings regarding CRE [37]. Furthermore, supplementation with LCA through rectal administration showed anti‐inflammatory effects that were partly reliant on TGR5 signaling in murine colitis models [38]. The potential mechanism might be associated with the promotion of intestinal epithelial regeneration by BAs through the activation of TGR5 in intestinal stem cells [39]. Owing to variations in populations, samples, and detection methods, no specific microbiota has been consistently associated with IBD. Nevertheless, intestinal dysbiosis is a consistently observed phenomenon, with the majority of studies indicating a reduction in microbial diversity among IBD patients [40]. The reduction of SCFA‐producing bacteria represents one of the common alterations [21], which aligns with our observations in CRE patients.

There were some limitations to this study. First, the single‐center study design might have introduced selection bias and limited the generalizability of our results. Second, the small sample size and high heterogeneity in the included participants might have affected the reliability of the BA and microbiota profiles. Third, as a retrospective study, validation through prospective, multicenter, large‐scale investigations is warranted to confirm our findings. Fourth, all patients in the CRE and NRE groups had undergone chemotherapy 3 months prior to enrollment. Therefore, we are unable to entirely exclude the influence of chemotherapy on gut microbiota. Despite these limitations, our study provides preliminary evidence of an association between fecal BAs and intestinal microbiota in patients with CRE. Future studies shall focus on further elucidation of the underlying mechanisms of this association.

In conclusion, patients with CRE exhibit dysregulated BA metabolism, characterized by an increased abundance of primary BAs and a decreased abundance of secondary BAs, particularly LCA and its isomer, when compared with the CC group. In addition, the abundance of beneficial bacterial genera, such as *Bifidobacterium* and *Megasphaera*, is reduced, whereas that of potentially pathogenic genera, including *Megamonas* and *Dorea*, is increased. Furthermore, a bidirectional relationship exists between BA metabolism and gut microbial composition. This suggests that a correlation may exist between fecal BAs and intestinal microbiota in patients with CRE, which may enhance our understanding of the etiology of CRE and suggest that interventions targeting BA metabolism and modulating intestinal microbiota may represent a promising therapeutic strategy for this condition.

## Funding

This study was supported by grants from the National Natural Science Foundation of China (nos. 82330100 and 82200567), the Healthcare Innovation Capability Enhancement Plan in Shaanxi Province (no. 2024TD‐06), and the Booster Plans of Xijing Hospital (no. JSYXM04).

## Conflicts of Interest

The authors declare no conflicts of interest.

## Supporting information


**Figure S1:** Altered bile acid (BA) profiles in the cervical cancer (CC), chronic radiation enteritis (CRE), and non‐CRE (NRE) groups. (a) Stacked bar chart of the relative proportions of primary and secondary BAs in each group. (b) PLS‐DA score plot of CC, CRE, and NRE groups. (c) *Z*‐score dot plot of the 31 differential BAs in CRE. *p < 0.05, **p < 0.01.

**Figure S2:** Permutation test (999 times) of the orthogonal partial least squares discriminant analysis (OPLS‐DA) models. (a) OPLS‐DA score plot of the chronic radiation enteritis (CRE) and cervical cancer (CC) groups. (b) OPLS‐DA score plot of the CRE and non‐CRE (NRE) groups. (c) OPLS‐DA model in (a): R^2^Y = 0.561, Q^2^Y = 0.26. (d) OPLS‐DA model in panel (b): R^2^Y = 0.818, Q^2^Y = 0.271.

**Figure S3:** Analysis of α‐diversity and β‐diversity of fecal microbiota in the chronic radiation enteritis (CRE), non‐CRE (NRE), and cervical cancer (CC) groups. (a) The Observed_species, Simpson, and Chao1 indexes between the case group and the control groups. (b) Principal coordinate analysis (PCoA) plot of β‐diversity of fecal microbiota in the three groups. (c) Non‐metric multidimensional scaling (NMDS) plot of β‐diversity analysis of fecal microbiota in the case and control groups.

**Table S1:** Concentrations of fecal bile acids (BAs) in the cervical cancer (CC), non‐chronic radiation enteritis (NRE), and chronic radiation enteritis (CRE) groups.

## Data Availability

The data that support the findings of this study are available from the corresponding author upon reasonable request.
