# Supplementary figures and images for: Relationship Between Fecal Bile Acid Profile and Intestinal Microbiota in Patients With Chronic Radiation Enteritis
Source: J Dig Dis. 2026 Jan 28;27(1-2):40–9. doi: 10.1111/1751-2980.70029 (PMC13097966; doi:10.1111/1751-2980.70029)

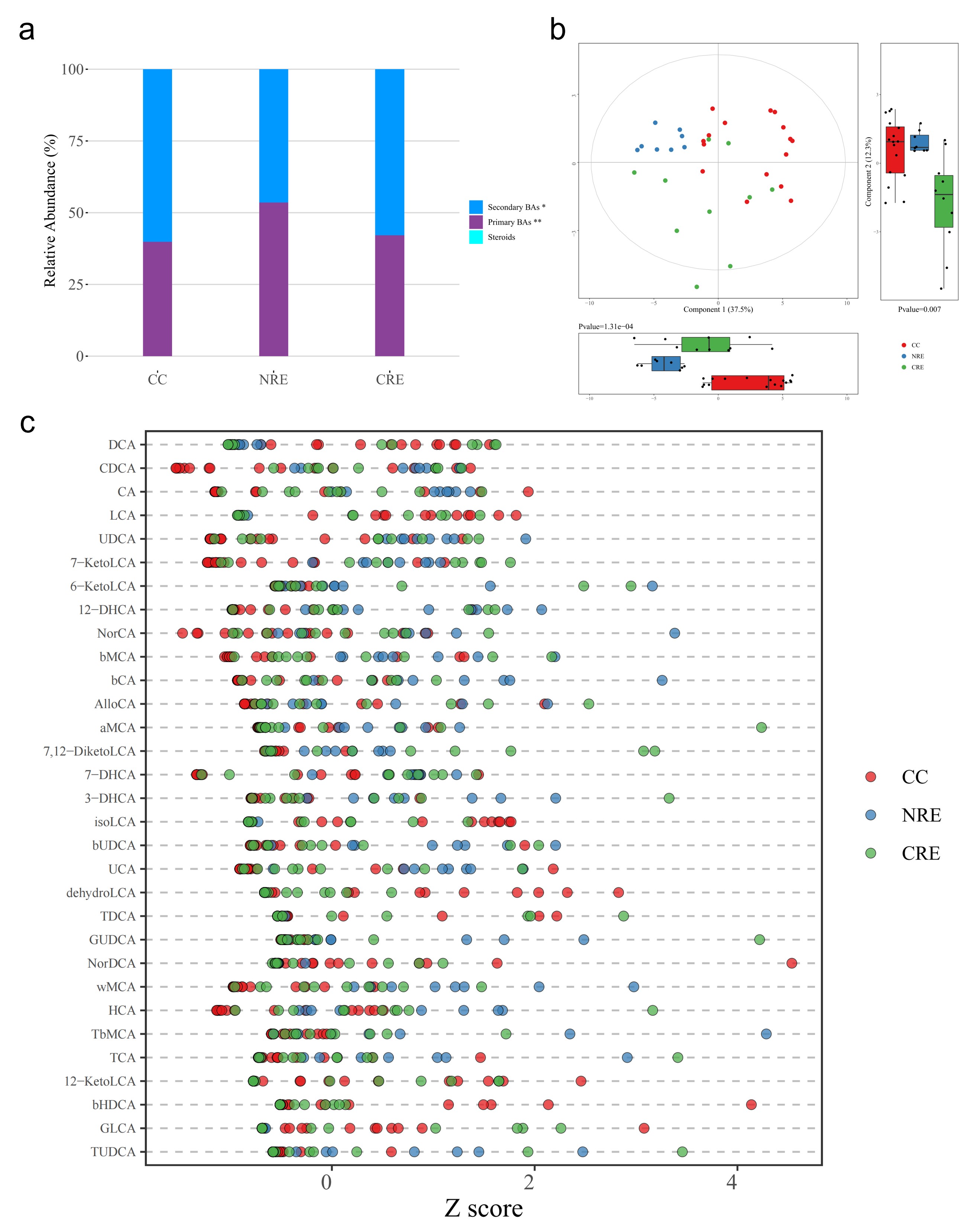

Supplement: Supplementary file 1 — Figure S1: Altered bile acid (BA) profiles in the cervical cancer (CC), chronic radiation enteritis (CRE), and non‐CRE (NRE) groups. (a) Stacked bar chart of the relative proportions of primary and secondary BAs in each group. (b) PLS‐DA score plot of CC, CRE, and NRE groups. (c) Z‐score dot plot of the 31 differential BAs in CRE. *p < 0.05, **p < 0.01. [file CDD-27-40-s001.jpg]

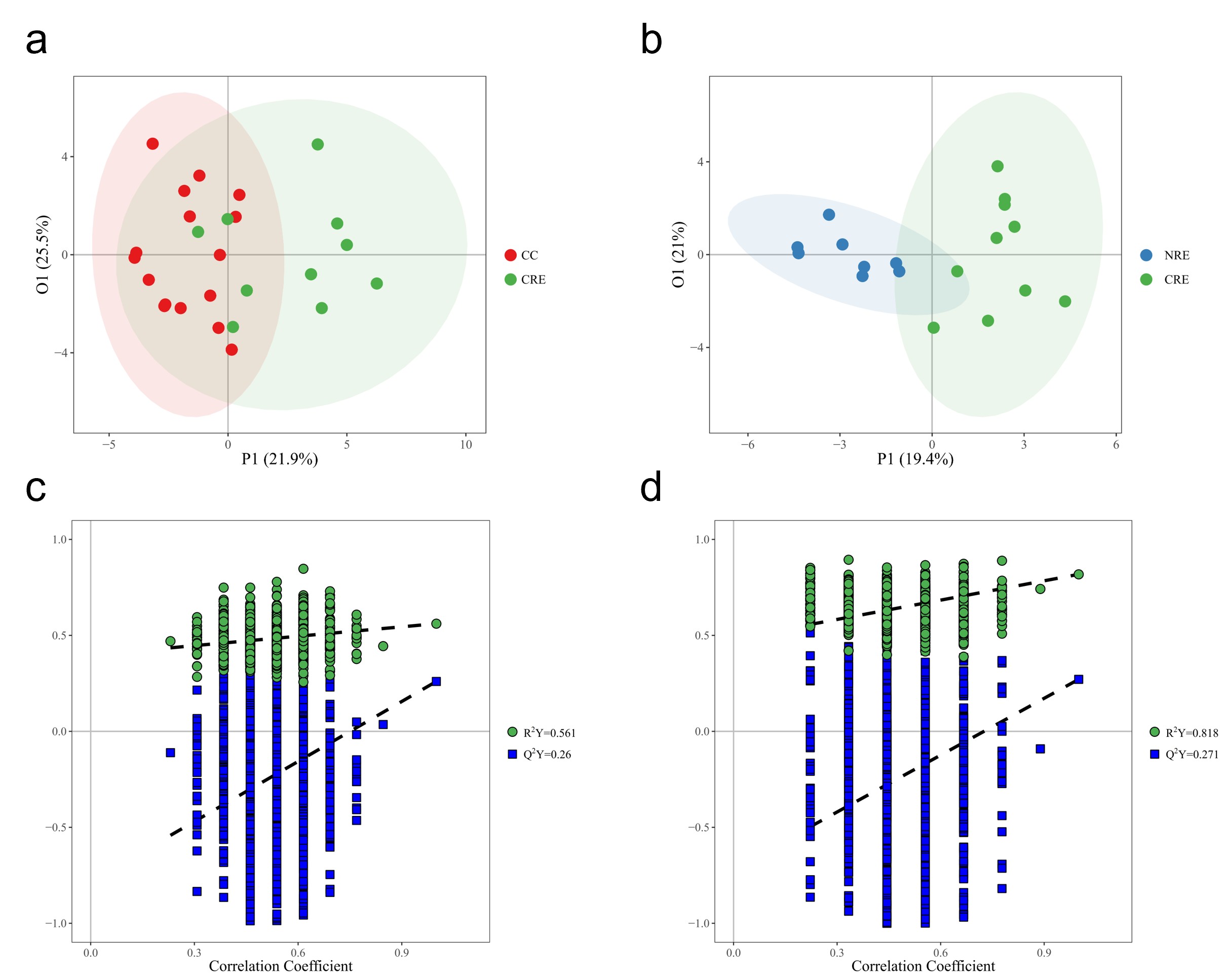

Supplement: Supplementary file 2 — Figure S2: Permutation test (999 times) of the orthogonal partial least squares discriminant analysis (OPLS‐DA) models. (a) OPLS‐DA score plot of the chronic radiation enteritis (CRE) and cervical cancer (CC) groups. (b) OPLS‐DA score plot of the CRE and non‐CRE (NRE) groups. (c) OPLS‐DA model in (a): R2Y = 0.561, Q2Y = 0.26. (d) OPLS‐DA model in panel (b): R2Y = 0.818, Q2Y = 0.271. [file CDD-27-40-s002.jpg]

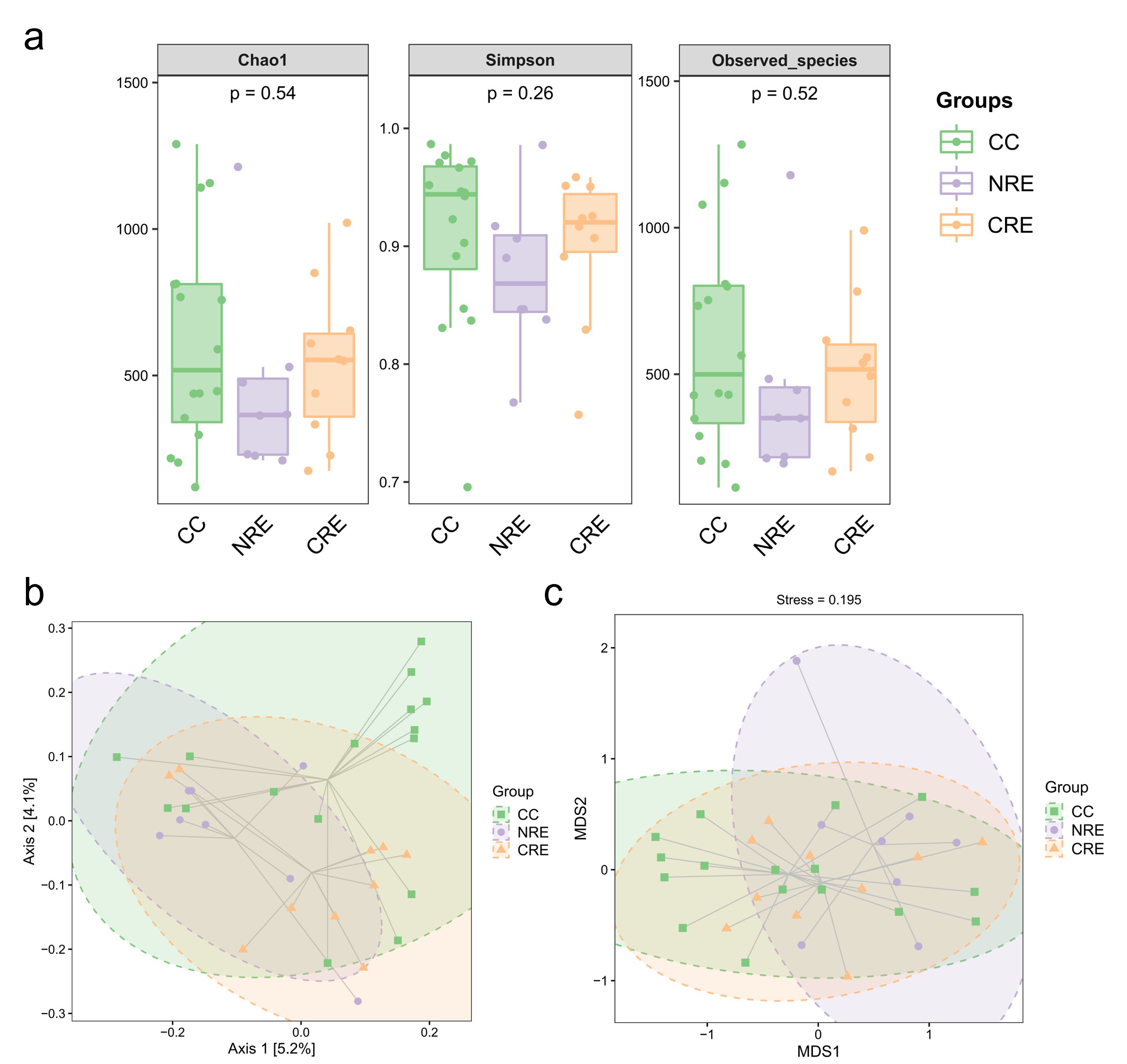

Supplement: Supplementary file 3 — Figure S3: Analysis of α‐diversity and β‐diversity of fecal microbiota in the chronic radiation enteritis (CRE), non‐CRE (NRE), and cervical cancer (CC) groups. (a) The Observed_species, Simpson, and Chao1 indexes between the case group and the control groups. (b) Principal coordinate analysis (PCoA) plot of β‐diversity of fecal microbiota in the three groups. (c) Non‐metric multidimensional scaling (NMDS) plot of β‐diversity analysis of fecal microbiota in the case and control groups. [file CDD-27-40-s004.jpg]
